# Maternal Satisfaction and Associated Factors with Postcesarean Section Pain Management: A Cross-Sectional Study

**DOI:** 10.1155/2024/4885678

**Published:** 2024-08-02

**Authors:** Biruk Adie Admass, Fikadu Tadesse Diress, Demeke Yilkal Fentie, Nigussie Simeneh Endalew

**Affiliations:** ^1^ Department of Anesthesia School of Medicine College of Medicine and Health Sciences University of Gondar, P.O. Box: 196, Gondar, Ethiopia; ^2^ Department of Anesthesia College of Medicine & Health Sciences Bahir Dar University, Bahir Dar, Ethiopia

## Abstract

**Background:**

Maternal satisfaction with pain management after cesarean delivery serves as an indicator of the quality of care. Assessing the level of satisfaction with postcesarean delivery pain management is paramount for both the mother and the healthcare institution. This study aimed to assess maternal satisfaction with postcesarean section pain management and associated factors at the Comprehensive Specialized Hospital in Northwest, Ethiopia, in 2023.

**Methods:**

An institution-based cross-sectional study was conducted from April to June 2023, involving 424 study participants. A consecutive sampling method was utilized for participant selection. Data were entered into Epidata and then exported to SPSS version 25 for analysis. Descriptive and analytic statistics were employed. Both bivariable and multivariable logistic regression analyses were conducted to identify factors associated with maternal satisfaction regarding postcesarean section pain management. Variables with a *p* value of <0.2 in the bivariable analysis were included in the multivariable analysis. In the multivariable analysis, variables with a *p* value of <0.05 were considered statistically significant. Crude odds ratio and adjusted odds ratio with 95% confidence intervals were calculated to demonstrate the strength of the association.

**Results:**

A total of 424 participants were included in the study with a response rate of 97.8%. The overall maternal satisfaction with postcesarean section pain management was 80.2% (95% CI: 76.1%–83.8%). Urban residence, elective cesarean section, mild pain, two and above previous history of cesarean section, and waiting less than 30 minutes to get analgesia were associated with maternal satisfaction with postcesarean section pain management.

**Conclusion:**

The overall maternal satisfaction with postcesarean section pain management was promising. Urban residence, elective cesarean section, previous history of cesarean section, mild pain, and waiting less than 30 minutes to get analgesia were predictor factors for maternal satisfaction with postcaesarian section pain management. We recommend that the stakeholders should give attention to enhancing maternal satisfaction.

## 1. Introduction

Cesarean section is one of the most common and important surgeries performed when vaginal birth poses a health risk to the mother or fetus [1]. The global cesarean section (CS) rate was 18.6% on average; however, rates in the least and most developed regions range from 6.0 to 27.2%, respectively [2]. In Ethiopia, the recent overall rate of cesarean deliveries was 29.55% [3].

Pain is the most common complaint among women having a cesarean section [4]. A study conducted at the University of Gondar Comprehensive Specialized Hospital reported that 85.5% of parturient delivered by cesarean section experienced moderate to severe postoperative pain in the first 24 postoperative hours [5]. If postcesarean pain is not managed properly, it can put mothers into a period of depression, affecting mother-child bonding and delaying recovery and returning to normal day-to-day activities [6]. Early recovery is important especially for a mother who has delivered via cesarean section to take care of her newborn shortly after an operative procedure [7].

Patient satisfaction is an essential component for assessing the quality of care and evaluating performance [8]. High satisfaction with healthcare is considered a desired outcome and may influence decisions to seek care, change providers or medical plans, and adhere to prescribed treatment plans [9].

If accurately measured, maternal satisfaction with postcesarean pain management can be a useful indicator of the quality of care in healthcare settings [10]. Health personnel play a major role in perioperative patient care, including assessment and treatment of postoperative pain [11].

Effective pain management depends not only on medication choices but also on the patient's expectations for pain management, which in turn influences satisfaction [12]. Satisfaction with pain management is the result of contentment with the care process and outcomes, including waiting time, access, and adequacy of care [13]. In healthcare settings, patient satisfaction typically includes both psychosocial and technical components of care, both of which are closely related to efficient pain management [14].

Despite the high number of cesarean births, no studies have been conducted to ascertain maternal satisfaction with pain management after cesarean section in our hospital. Therefore, this study aimed to assess maternal satisfaction with postcesarean section pain management and its associated factors at our institution.

## 2. Methods

### 2.1. Design and Setting

This hospital-based cross-sectional study was conducted between April and June 2023 at the University of Gondar Comprehensive Specialized Hospital, located in the Amhara regional state in Northwest Ethiopia. The hospital serves as a teaching and comprehensive referral healthcare institution. The hospital has two rooms for emergency cesarean section and one room for elective cesarean section. The hospital has 14 beds for the admission of pregnant women for elective cesarean section and 24 beds for emergency elective cesarean section. Data from the hospital operation register logbook indicated that around 4500–5000 cesarean deliveries were done annually. During the study period, a total of 1054 elective and emergency cesarean sections were performed. We included women who underwent caesarean section and stayed for 24 hours after cesarean section. Mothers under the age of 18 years, patients with lower levels of consciousness, and those unwilling to provide informed consent for the study were excluded from participation.

In our setting, unless contraindicated, spinal anesthesia is the preferred anesthetic choice, administered with 1.8–2 ml of 0.5% bupivacaine alone or with the addition of 25 *μ*g of fentanyl or 25 mg of pethidine to enhance analgesia for cesarean sections. Following surgery, our standard postoperative analgesic regimen includes an abdominal field block (Transversus abdominis plane block with 15−20 ml of 0.25% bupivacaine bilaterally), followed by systemic analgesic medication (50−100 mg of IV tramadol or 75 mg of IM diclofenac) as needed based on pain assessment using the numerical rating scale.

### 2.2. Study Variables

The outcome variable of this study was maternal satisfaction with pain management after a cesarean section. The independent variables were sociodemographic, clinical, and analgesic-related factors.

Maternal satisfaction with postoperative pain management after cesarean section was measured via a 5-point Likert scale. The maternal satisfaction scale was dichotomized into satisfied and dissatisfied groups based on the demarcation threshold formula (highest value-lower value/2) + lower value [15–17]. Mothers who scored less than 28 out of 45 were considered dissatisfied, whereas those who scored 28 and above were considered satisfied.

Neonatal outcome is categorized and defined using the APGAR score, which stands for appearance (skin color), pulse (heart rate), grimace (reflex irritability), activity (muscle tone), and respiration. This scoring system provides a quick assessment of the health of all neonates at 1 and 5 minutes after birth and in response to resuscitation efforts. A neonate is considered “alive and well” if they have an APGAR score of 7–10. Conversely, a neonate with an APGAR score of less than 7 is categorized as live but ill.

### 2.3. Sample Size Determination

The sample size of the study was determined by using a single population proportion formula. The size of the study participants was calculated using a 95% level of confidence and a 5% margin of error. No similar study was conducted in Ethiopia to assess maternal satisfaction with postoperative pain management after cesarean delivery. Thus, the 0.5 proportion assumption was applied. With a 10% nonresponse rate, the final sample size was 424.

### 2.4. Sampling Technique

A consecutive sampling technique was used to select the study participant. Participants who fulfilled the criteria were included in the study until the required sample size was achieved.

### 2.5. Data Collection and Quality Control

Data were collected 24 hr after the operation through chart review and interviews. The questionnaire was translated into the local language (Amharic). The questionnaire had the following four sections: sociodemographic characteristics, clinical-related factors, analgesia-related factors, and the Pain Treatment Satisfaction Scale (PTSS). PTSS is a valid assessment tool for pain treatment satisfaction with Cronbach's alpha of 0.87 [18]. The PTSS has the following five dimensions: information on pain, pain medication, medical care, impact of current pain medication, side effects of pain medication, and satisfaction with pain medication and care. The first four dimensions were rated on a five-point Likert scale. Only the side effects of pain medication were rated on a six-point Likert scale (0 = no experience and 5 = extremely bothered). The last subsection of the Pain Treatment Satisfaction Scale (PTSS), which assesses satisfaction with pain treatment and care, was used to assess the respondents' satisfaction with pain management [19–21]. The nine items are rated on a five-point Likert scale (1 = very dissatisfied and 5 = very satisfied).

To ensure the quality of data, a pretest was conducted on 5% of the mothers after cesarean section. The accuracy, completeness, and consistency of the data collection were cross-checked before the analysis.

### 2.6. Data Analysis and Interpretation

The data were entered using Epidata version 4.6 and then exported to SPSS version 25 for data analysis. Descriptive statistics were performed, and the results were presented using text, tables, and graphs. Multicollinearity among independent variables was assessed using the variance inflation factor (VIF) and a tolerance test. The goodness of fit of the logistic regression model was evaluated using the Hosmer–Lemeshow test, which indicated a good fit. The normality of continuous variable distributions was assessed using the Shapiro–Wilk test.

A binary logistic regression analysis model was employed to determine the presence of statistically significant associations between maternal satisfaction with postcesarean section pain management and independent variables, with a 95% confidence interval (CI). All variables with a *p* value <0.2 in the bivariable analysis were included in the multivariable logistic regression analysis, and significance was determined at a *p* value <0.05. The strength of association between dependent and independent variables was assessed using the adjusted odds ratio [22].

### 2.7. Ethics Approval and Consent to Participate

Ethical approval was obtained from the Ethical Review Board of the School of Medicine, College of Medicine and Health Sciences, University of Gondar, with registration number SOM 06/01/584/2023. Written informed consent was obtained from each participant, and confidentiality was maintained at all levels of the study.

## 3. Results

### 3.1. Sociodemographic Characteristics of the Respondents

A total of 424 participants were recruited with a 97.8% (415) response rate. Nine participants were excluded from the analysis due to incomplete data. The age range of 26–33 years old comprised the majority of participants (50.6%), with a median age of 28. Of the 216 participants, 52% were from rural locations (Table 1).

### 3.2. Clinical Characteristics of the Respondents

**Figure 1:**
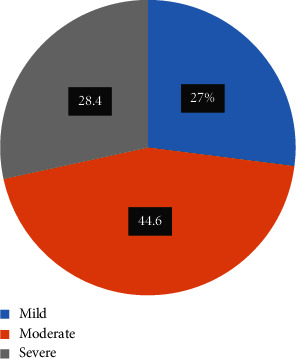
Pain intensity after postcesarean section within 24 hr at University of Gondar Comprehensive Specialized Hospital in Northwest Ethiopia, 2023.

315 (75.9%) of the 415 study participants had emergency caesarean section. Spinal anesthesia was used in the majority of the participants, i.e., 405 (97.6%). About 311 participants (74.9%) had never undergone a cesarean section before (Table 2). Within 24 hours, the majority of participants (44.6%) reported moderate pain, while 27% and 28.1% reported mild and severe pain, respectively (Figure 1).

### 3.3. Analgesia-Related Characteristics of the Respondents

**Figure 2:**
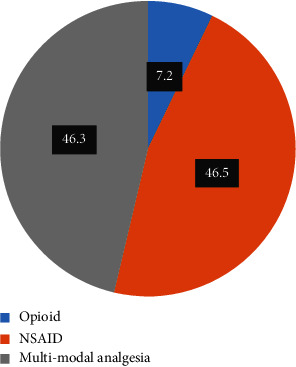
Type of analgesia given postcesarean section at University of Gondar Comprehensive Specialized Hospital in Northwest Ethiopia, 2023.

Of the participants involved, 192 (46.3%) had received multimodal analgesia (Figure 2). The percentage of participants who obtained analgesia within 30 minutes of requesting pain medication was higher (284, 68.4%) (Table 2).

### 3.4. Pain Treatment Satisfaction Scale

The majority of participants preferred to have information about their injury or illness and understood the cause of their pain. In addition, the majority of mothers (86.1%) indicated a preference to know nothing about potential adverse effects of analgesics. Conversely, only 4.8% of the participants expressed a preference for substantially more information regarding their pain treatment options (Table 3).

In response to medical care questions, more than half of the participants somewhat agreed that it was easy to ask questions to medical staff (*n* = 220, 53%). Nearly half of the mothers believed that medical staff were willing to provide pain medication that they felt they needed (*n* = 205, 49.4%). A greater number of participants strongly agreed that medical staff provided adequate follow-up care (*n* = 42, 10.1%) (Table 4).

Most participants either agreed or strongly agreed with all statements regarding pain medication, improving their health, function, participation, mood, and cognition (Table 5). However, most participants did not experience these negative side effects. Some participants experienced bothersome side effects including nausea, vomiting, excessive fatigue, and inability to concentrate (Table 6).

### 3.5. Satisfaction Related to Postcesarean Section Pain Management

**Figure 3:**
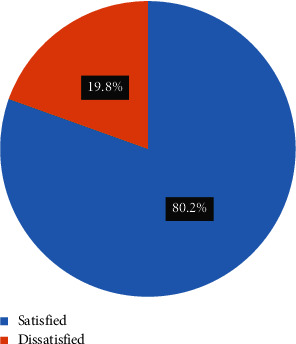
The overall maternal satisfaction on postcesarean pain management at University of Gondar Comprehensive Specialized Hospital, Northwest Ethiopia, 2023.

In this study, satisfaction with postcesarean section pain management was dichotomized as satisfied and dissatisfied. Nine items of the PTSS were used to measure maternal satisfaction (Table 7). Using a demarcation threshold method, scores between 28 and above indicated satisfaction and scores below 28 indicated dissatisfaction. Overall, 80.2% (CI: 76.1%–83.8%) of the participants expressed satisfaction with the pain management following a cesarean section (Figure 3).

### 3.6. Factors Associated with Maternal Satisfaction on Postcesarean Section Pain Management

Maternal satisfaction with postcesarean section pain treatment was found to be associated with several factors in bivariate logistic regression analysis. These factors included residence, monthly income, urgency of operation, pain severity, neonatal outcome, number of previous cesarean sections, and waiting time to obtain analgesia.

In multivariate binary logistic regression analysis, certain factors were identified as significantly associated with maternal satisfaction with postcesarean section pain management. Urban residence, elective surgery, mild pain intensity, two or more previous cesarean sections, and waiting for less than 30 minutes to receive analgesia were significant of maternal satisfaction with postcesarean section pain management (Table 8).

Participants living in urban areas were 1.7 times more satisfied with postcaesarean section pain management than those in rural areas (AOR = 1.799, 95% CI: 1.064–3.042). The odds of maternal satisfaction with postcesarean section pain management were 2.2 times higher in the elective procedure group compared to the emergency group (AOR = 2.227, 95% CI: 1.1–4.511) (Table 8).

Maternal satisfaction with postcesarean section pain management was shown to be 2.7 times greater in those who had two or more previous cesarean sections than in those who did not (AOR = 2.766, 95% CI: 1.023–7.479). When analgesia was obtained quickly (less than 30 minutes), maternal satisfaction with postcesarean pain management was 1.8 times higher than when it took longer (more than 30 minutes) to obtain analgesia (AOR = 1.811, 95% CI: 1.073–3.057) (Table 8).

## 4. Discussion

In this study, the overall results showed that 80.2% participants were satisfied with postcesarean section pain management in the first 24 hours. These results were lower compared to other studies reported in Pakistan (91.6%), Kenya (85%), and Jordan (95%) [23–25]. The presence of institutional pain treatment policies, as described in these studies, engagement of anesthesiologists in pain management, and variations in evaluation techniques could be the cause of this discrepancy.

However, this finding was higher than the results of other studies conducted in Rwanda (76.1%), Uganda (68.0%), and Nigeria (60%) [26–28]. The inconsistent and insufficient availability of painkillers was cited as one explanation for this study's comparatively low satisfaction rate in Uganda. Consequently, the patients had to purchase their own drugs during the data collection period, as stated in the study [28].

Regarding the factors associated with maternal satisfaction with postcesarean section pain management, five variables were statistically identified. The first factor that was significantly associated with satisfaction with postcesarean section pain management was women who had elective versus emergency cesarean section. This finding is supported by studies conducted in the USA and Germany. They stated that satisfaction with pain relief was provided following elective caesarean section with optimum pain management [29]. Parturient of elective caesarean deliveries are psychologically prepared and counseled over time.

In the present study, urban residence was found to be significantly associated with satisfaction with postcesarean section pain management compared to rural residences. These findings are consistent with a study conducted in Arba Minch, which also reported an increased likelihood of satisfaction among patients from urban residences compared to those from rural residences [30].

In contrast, a study conducted in US reported that compared to urban areas, rural areas were significantly more likely to be satisfied with pain management [31]. This variation may be due to access to information, cultural differences, and unrealistic expectations regarding care appropriateness.

The current study also found an association between maternal satisfaction with postcesarean section pain management and having undergone two or more cesarean sections in the past. This result was in line with a study conducted in Mexico which indicated a positive correlation between the number of cesarean sections performed and maternal satisfaction with pain management [32]. This indicates that women who had a higher number of cesarean sections reported statistically significant higher satisfaction on postcesarean section pain management. This might be due to their prior personal experiences, knowledge, and expectations of postsurgical pain as well as the relative change in pain from presurgical to postsurgical levels.

According to this study, there was a significant association between pain severity and maternal satisfaction with pain management after cesarean section. Compared to those with severe pain, those with mild pain were more satisfied with postcesarean section pain management. This finding supported by other studies in which women who experienced severe pain had lower satisfaction than those who experienced moderate pain [33, 34]. This may be influenced by patient expectations, individual pain thresholds, and effectiveness of pain management interventions.

This study also found an association between the amount of time she had to wait to receive analgesia and maternal satisfaction with postcesarean section pain management. Patients with less than 30 min of waiting time to receive analgesia had better satisfaction with postcesarean section pain management than those with a waiting time of 30 min or longer to obtain analgesia. This finding is consistent with that of a study done in Tikur Anbessa Comprehensive Specialized Hospital in Ethiopia. They reported that waiting for a short time (<30 min) to receive analgesics to pain was positively associated with satisfaction [35]. This finding is consistent with other study where patients expressed dissatisfaction with pain management when experiencing longer waiting times for medication [36]. Receiving timely pain relief can significantly improve the overall experience of patients, making them feel more comfortable.

### 4.1. Limitation of the Study

While this study is considered as an important healthcare quality indicator in assessing maternal satisfaction with postcesarean section pain management, it has some drawbacks. This study focused primarily on the outcome of acute pain treatment in terms of maternal satisfaction overlooks an examination of the broader pattern of pain management. The utilization of consecutive sampling techniques may have a risk of committing selection bias. Moreover, the study was conducted in a single-center setting and used intrathecal pethidine as an adjuvant to spinal anesthesia, which may affect the generalizability of its findings.

## 5. Conclusion and Recommendation

The overall score for maternal satisfaction with postcesarean section pain management was promising. The study identified several factors significantly associated with maternal satisfaction, including urban residence, elective surgery, two or more previous cesarean sections, mild pain intensity, and shorter waiting time (<30 min) for analgesia administration.

Clinicians should prioritize these factors when aiming to enhance maternal satisfaction with postcesarean section pain management. Particular attention should be directed towards mothers residing in rural areas and those undergoing emergency cesarean sections. In addition, it is crucial to conduct thorough assessments of pain severity when devising pain management interventions. Healthcare providers should prioritize efforts to minimize waiting times for receiving analgesia. Furthermore, special attention should be given to clients undergoing cesarean sections for the first time, aiming to enhance their satisfaction with pain management aspects.

## Figures and Tables

**Table 1:** Sociodemographic characteristics of participants who received pain management at University of Gondar Comprehensive Specialized Hospital in Northwest Ethiopia (*n* = 415).

Variable		Frequency	Percentage
Age	18–25	114	27.5
	26–33	210	50.6
	34–41	91	21.9

Residency	Urban	199	48
	Rural	216	52

Marital status	Unmarried	9	2.2
	Married	406	97.8

Education	No formal education	147	35.4
	Primary education	138	33.3
	Secondary education	99	23.9
	Higher education	31	7.5

Monthly income	<1500	72	17.4
	1500–5000	220	53.0
	>5000	123	29.6

Occupation	Unemployed	115	27.7
	Self employed	220	53
	Government worker	80	19.3

**Table 2:** Clinical characteristics of postcesarean section mothers who received pain management at University of Gondar Comprehensive Specialized Hospital in Northwest Ethiopia, 2023 (*n* = 415).

Variable		Frequency	Percentage
Urgency of surgery	Emergency	315	75.9
	Elective	100	24.1

Type of anesthesia	Spinal anesthesia	405	97.6
	General anesthesia	10	2.4

Previous CS history	None	311	74.9
	One previous history	59	14.3
	Two and above previous histories	45	10.8

Neonatal outcome	Alive and well	392	94.5
	Alive and ill	23	5.5

Number of gestations	Single tone	401	96.6
	Twin and above	14	3.4

Waiting time for pain medication	Within 30 minutes	284	68.4
	30 minutes and later	131	31.6

**Table 3:** Information about pain medication.

How much information would you have liked to have received about each of the following	I would have liked no information	I would have liked less information	The amount of information was right for me	I would have liked little more information	I would have liked much more information
My illness or injury	0	142 (34.2%)	128 (30.8%)	97 (23.4%)	48 (11.6%)
The cause(s) of my pain	0	146 (35.2%)	135 (32.5%)	91 (21.9%)	43 (10.4%)
Treatment option for my pain	124 (29.9%)	120 (28.9%)	89 (21.4%)	61 (14.7%)	21 (5.1%)
Pain medication in general	240 (57.8%)	73 (17.6%)	64 (15.4%)	31 (7.5%)	7 (1.7%)
Possible side effect of pain medication	357 (86.1%)	45 (10.8%)	9 (2.2%)	3 (0.7%)	1 (0.2)

**Table 4:** Medical care.

How much do you agree or disagree with each of the following statement	Strong disagree	Somewhat disagree	Neither agree or disagree	Somewhat agree	Strongly agree
It is easy to ask questions for medical staff	22 (5.3%)	48 (11.6%)	90 (21.7%)	220 (53%)	35 (8.4%)
The medical staff always do their best to keep me from worrying	25 (6%)	47 (11.3%)	100 (24.1%)	204 (49.2%)	39 (9.4%)
The medical staff is willing to provide me with the pain medication that I feel I need	24 (5.8%)	45 (10.8%)	105 (25.3%)	205 (49.4%)	36 (8.7%)
The medical staff provide adequate follow-up care	27 (6.5%)	57 (13.7%)	99 (23.9%)	190 (45.8%)	42 (10.1%)
The medical staff does not ask me about the pain I experience	30 (7.2%)	86 (20.7%)	207 (49.9%)	76 (18.3%)	16 (3.9%)

**Table 5:** Current pain medication.

How much do you agree or disagree with each of the following statement	Strongly disagree	Disagree	Neither agree or disagree	Agree	Strongly agree
My pain medication has a positive effect on my physical health	13 (3.1%)	21 (5.1%)	107 (25.8%)	212 (51.1%)	62 (14.9%)
My pain medication helps me have a better outlook on life	9 (2.2%)	26 (6.2%)	133 (32%)	199 (48%)	48 (11.6%)
My pain medication allows me to perform my daily activity more easily	12 (2.9%)	23 (5.5%)	133 (32.1%)	203 (48.9%)	44 (10.6%)
My pain medication allows me to participate in my leisure activity more often	16 (3.9%)	25 (6%)	156 (37.6%)	172 (41.4%)	46 (11.1%)
My pain medication helps me do things independently	22 (5.3%)	26 (6.3%)	178 (42.9%)	152 (36.6%)	37 (8.9%)
My pain medication allows me to have better relationship with other	10 (2.4%)	20 (4.8%)	166 (40%)	170 (41%)	49 (11.8%)
My pain medication improves my mood	3 (0.7%)	4 (1%)	103 (24.8%)	216 (52.1%)	89 (21.4%)
My pains medication allows me to concentrate better	5 (1.2%)	7 (1.7%)	138 (33.3%)	198 (47.7%)	67 (16.1%)

**Table 6:** Side effect of pain medication.

Because of pain medication how much were you bothered by the following	Did not experience	Not bother at all	A little bothered	Moderately bothered	Quite bothered	Extremely bothered
Unintentional weight gain	0	0	0	0	0	0
Excessive fatigue	412 (99.3%)	3 (0.7%)	0	0	0	0
Drowsiness	408 (98.3%)	7 (1.7%)	0	0	0	0
Inability to concentrate	413 (99.5%)	2 (0.5%)	0	0	0	0
Nausea	352 (84.8%)	26 (6.3%)	16 (3.9%)	13 (3.1%)	5 (1.2%)	3 (0.7%)
Diarrhea	415 (100%)	0	0	0	0	0
Dizziness	415 (100%)	0	0	0	0	0
Constipation	415 (100%)	0	0	0	0	0
Skin rash	414 (99.8%)	1 (0.2)	0	0	0	0
Stomach aches	415 (100%)	0	0	0	0	0
Heartburn	415 (100%)	0	0	0	0	0
Vomiting	384 (92.6%)	20 (4.8%)	3 (0.7%)	3 (0.7%)	5 (1.2%)	0

**Table 7:** Satisfaction to pain treatment and care.

How satisfied are you with each of the following	Very dissatisfied	Dissatisfied	Neither satisfied nor dissatisfied	Satisfied	Very satisfied
The information that you received about your pain and treatment	63 (15.2%)	56 (13.5%)	97 (23.4%)	160 (38.5%)	39 (9.4%)
The amount of time that the doctor devoted to you during their visit/consultation	51 (12.3%)	75 (18%)	121 (29.2%)	130 (31.3%)	38 (9.2%)
The care provided by the nurses for your pain and its treatment	25 (6%)	42 (10.1%)	125 (30.1%)	160 (38.6%)	63 (15.2)
The form of medication (pill, capsule, injection)	5 (1.2%)	15 (3.6%)	77 (18.6%)	244 (58.8%)	74 (17.8%)
How often you take your medication	10 (2.4%)	25 (6.1%)	103 (24.8%)	211 (50.8%)	66 (15.9%)
The amount of pain medication you take	6 (1.4%)	28 (6.7%)	134 (32.3%)	180 (43.4%)	67 (16.2%)
The level or amount of pain relief provided by your pain medication	6 (1.4%)	34 (8.2%)	133 (32%)	171 (41.3%)	71 (17.1%)
The duration of pain relief provided by your pain medication	23 (5.6%)	49 (11.8%)	123 (29.6%)	164 (39.5%)	56 (13.5%)
The time that it takes your pain medication to work	4 (1%)	7 (1.7%)	52 (12.6%)	231 (55.6%)	121 (29.1%)

**Table 8:** Factors associated with maternal satisfaction on postcesarean section pain management at University of Gondar Comprehensive and Specialized Hospital, Northwest Ethiopia, 2023.

Variable	Categories	Satisfied *n* (%)	Dissatisfied *n* (%)	COR (95%)	AOR	*P* value
Residence	Urban	168 (84.4%)	31 (15.6%)	1.67 (1.02–2.74)	1.799 (1.064–3.042)	0.028
	Rural	165 (76.4%)	51 (23.6%)	1	1	

Monthly income	<1500ETB	53 (73.6%)	19 (26.4%)	1	1	
	1500–5000	175 (79.5%)	45 (20.5%)	1.39 (0.75–2.58)	1.207 (0.626–2.326)	0.575
	≥5000	105 (85.4%)	18 (14.6%)	2.09 (1.01–4.31)	1.475 (0.683–3.186)	0.322

Urgence of surgery	Elective	89 (89%)	11 (11%)	2.35 (1.19–4.64)	2.227 (1.1–4.511)	0.026
	Emergency	244 (77.5%)	71 (22.5%)	1	1	

Pain intensity	Mild	97 (86.6%)	15 (13.4%)	2.30 (1.16–4.55)	2.079 (1.015–4.258)	0.045
	Moderate	149 (80.5%)	36 (19.5%)	1.47 (0.85–2.55)	1.533 (0.855–2.749)	0.152
	Severe	87 (73.7%)	31 (26.3%)	1	1	

Neonatal outcome	Alive and well	318 (81.1%)	74 (18.9%)	2.29 (0.93–5.60)	1.79 (0.695–4.614)	0.228
	Alive and ill	15 (65.2%)	8 (34.8%)	1	1	

Number of previous history C/S	None	242 (77.8%)	69 (22.2%)	1	1	
	One previous	51 (86.4%)	8 (13.6%)	1.81 (8.82–4.01)	1.672 (0.726–3.848)	0.227
	Two and above previous	40 (88.9%)	5 (11.1%)	2.28 (0.86–6.00)	2.766 (1.023–7.479)	0.045

Waiting time to get analgesia	<30 minute	238 (83.8%)	46 (16.2%)	1.96 (1.19–3.22)	1.811 (1.073–3.057)	0.026
	≥30 minute	95 (72.5%)	36 (27.5%)	1	1	

1 = reference group, COR = crude odd ratio, CI: confidence interval, AOR: adjusted odd ratio.

## Data Availability

The datasets used and/or analyzed during the current study are available from the corresponding author upon reasonable request.
