# Corrigendum to “Maternal Satisfaction and Associated Factors with Postcesarean Section Pain Management: A Cross-Sectional Study”

**DOI:** 10.1155/anrp/9838065

**Published:** 2025-08-21

B. A. Admass, F. T. Diress, D. Y. Fentie, and N. S. Endalew, “Maternal Satisfaction and Associated Factors with Postcesarean Section Pain Management: A Cross-Sectional Study,” *Anesthesiology Research and Practice* 2024 (2024): 4885678, https://doi.org/10.1155/2024/4885678.

In the article titled “Maternal Satisfaction and Associated Factors with Postcesarean Section Pain Management: A Cross-Sectional Study,” author order in the author list was incorrect, where

Biruk Adie Admass,^1^ Fikadu Tadesse Diress,^2^ Demeke Yilkal Fentie,^1^ and Nigussie Simeneh Endalew^1^

^1^Department of Anesthesia, School of Medicine, College of Medicine and Health Sciences, University of Gondar, P.O. Box: 196, Gondar, Ethiopia

^2^Department of Anesthesia, College of Medicine & Health Sciences, Bahir Dar University, Bahir Dar, Ethiopia

Should have read:

Fikadu Tadesse Diress,^1^ Demeke Yilkal Fentie,^2^ Nigussie Simeneh Endalew,^2^ and Biruk Adie Admass^2^

^1^Department of Anesthesia, College of Medicine & Health Sciences, Bahir Dar University, Bahir Dar, Ethiopia

^2^Department of Anesthesia, School of Medicine, College of Medicine and Health Sciences, University of Gondar, P.O. Box: 196, Gondar, Ethiopia

We apologize for this error.

